# Luteolin Protects Against 6-Hydoroxydopamine-Induced Cell Death *via* an Upregulation of HRD1 and SEL1L

**DOI:** 10.1007/s11064-023-04019-2

**Published:** 2023-08-26

**Authors:** Hiroki Nishiguchi, Tomohiro Omura, Ayaka Sato, Yumi Kitahiro, Kazuhiro Yamamoto, Junichi Kunimasa, Ikuko Yano

**Affiliations:** 1 Department of Pharmacy, Kobe University Hospital, 7-5-2 Kusunoki-cho, Chuo-ku, Kobe, 650-0017 Japan; 2 Education and Research Center for Clinical Pharmacy, Kobe Pharmaceutical University, 4-19-1, Motoyama Kitamachi, Higashinada-ku, Kobe, 658-8558 Japan

**Keywords:** luteolin, Parkinson’s disease, endoplasmic reticulum stress, HRD1, SEL1L

## Abstract

Parkinson’s Disease (PD) is caused by many factors and endoplasmic reticulum (ER) stress is considered as one of the responsible factors for it. ER stress induces the activation of the ubiquitin-proteasome system to degrade unfolded proteins and suppress cell death. The ubiquitin ligase 3-hydroxy-3-methylglutaryl-coenzyme A reductase degradation 1 (HRD1) and its stabilizing molecule, the suppressor/enhancer lin-12-like (SEL1L), can suppress the ER stress *via* the ubiquitin-proteasome system, and that HRD1 can also suppress cell death in familial and nonfamilial PD models. These findings indicate that HRD1 and SEL1L might be key proteins for the treatment of PD. Our study aimed to identify the compounds with the effects of upregulating the HRD1 expression and suppressing neuronal cell death in a 6-hydroxydopamine (6-OHDA)-induced cellular PD model. Our screening by the Drug Gene Budger, a drug repositioning tool, identified luteolin as a candidate compound for the desired modulation of the HRD1 expression. Subsequently, we confirmed that low concentrations of luteolin did not show cytotoxicity in SH-SY5Y cells, and used these low concentrations in the subsequent experiments. Next, we demonsrated that luteolin increased HRD1 and SEL1L mRNA levels and protein expressions. Furthermore, luteolin inhibited 6-OHDA-induced cell death and suppressed ER stress response caused by exposure to 6-OHDA. Finally, luteolin did not reppress 6-OHDA-induced cell death when expression of HRD1 or SEL1L was suppressed by RNA interference. These findings suggest that luteolin might be a novel therapeutic agent for PD due to its ability to suppress ER stress through the activation of HRD1 and SEL1L.

**Supplementary Information:**

The online version contains supplementary material available at 10.1007/s11064-023-04019-2.

## Introduction

Parkinson’s disease (PD) is a movement disorder, and the second most frequent neurodegenerative disorder after Alzheimer’s disease [1]. In PD patients, the content of dopamine in the striatum is reduced due to a loss of dopamine neurons in substantia nigra pars compacta of the midbrain [2]. Dopaminergic drugs are commonly used as a symptomatic treatment for PD, but the long-term use of these drugs is accompanied by a number of adverse effects [3]. The endoplasmic reticulum (ER) stress has been recently identified as one of the potential causative factors of PD, and it has been associated with the dopaminergic neuronal death occurring in the substantia nigra [4, 5].

ER has important functions, including protein folding, synthesis and glycosylation; the loss of these functions by a variety of stresses can lead to the unfolded protein accumulation in the ER, a phenomenon termed ER stress, thereby resulting in cell death. In response to ER stress, eukaryotic cells trigger a physiological reaction that prevents the accumulation of folding proteins (the so called “unfolded protein response” or “UPR”) [6]. Through one of the UPR systems, the ER-associated degradation (ERAD), the unfolded proteins are retro-transported from the ER to the cytoplasm *via* translocons, and they are poly-ubiquitinated by ubiquitin-conjugating enzymes such as the E3 ubiquitin ligase and other component proteins. Ultimately, these poly-ubiquitinated unfolding proteins are degraded by the 26 S proteasome [7, 8].

3-Hydroxy-3-methylglutaryl-coenzyme A (HMG-CoA) reductase degradation 1 (HRD1) is a type of an E3 ubiquitin ligase. It is related to ERAD, and has been identified as a human homolog of the yeast’s Hrd1p [9]. Interestingly, the suppressor/enhancer Lin12 1-like (SEL1L) has also been identified as a human homolog of the yeast’s Hrd3p [10]. It has been reported that Hrd3p and SEL1L stabilize Hrd1p and HRD1, respectively [11, 12]. HRD1/SEL1L-mediated protein degradation is an important process for the suppression of cell death induced by ER stress in the mammalian cells [13, 14].

We have previously reported that HRD1 is localized in the dopamine neurons of the substantia nigra pars compacta of the mouse midbrain [15]. Additionally, HRD1 has been known to degrade the Parkin-associated endothelin receptor-like receptor (Pael-R; a substrate of the ubiquitin ligase Parkin) [16], thereby resulting in the suppression of the cell death induced by the accumulation of Pael-R [17]. In the case of autosomal recessive juvenile Parkinsonian patient, misfolded Pael-R accumulates in the ER, thereby causing an ER stress-induced cell death due to the disruption of Parkin’s function as a result of a gene mutation [16]. HRD1 is also known to alleviate neuronal cell death in PD models using 6-hydroxydopamine (6-OHDA), that are widely used for the experimental simulation of PD both in vitro and in vivo [18–21]; a fact suggesting that HRD1 is an important molecule for the pathogenesis of PD. We have also previously reported that knockdown of SEL1L by RNA interference or direct suppression of SEL1L expression by transfection of miR-101 (a microRNA mimic) also enhances cell death in an in vitro PD model, suggesting an important role for SEL1L in the pathogenesis of PD [20, 22].

Accordingly, the compounds that upregulate the HRD1 expression might have a possibility to prevent the induction of ER stress and suppress the pathogenesis of PD. This study aimed to identify the compounds that upregulate HRD1 by using a drug repositioning tool, and evaluate whether the compounds can suppress cell death *via* an activation of HRD1 in cellular models of PD.

## Methods and Materials

### Drug Discovery

The candidate compounds that upregulate HRD1 were investigated by using the drug repositioning tool Drug Gene Budger (DGB) [23]. DGB is a web-based instrument that returns small molecule compounds predicted to influence the expression of genes of interest maximally. Researchers can query the genes the expression of which they want to upregulate, and DGB produces in a ranked list of compounds that have been experimentally found to yield the desired expression effect. The experimental data of DGB was procured from the Library of Integrated Network-Based Cellular Signatures (LINCS) L1000 dataset [24], the Connectivity Map (Cmap) dataset [25], and the Gene Expression Omnibus (GEO) database [26, 27]. In this study, we selected the results from the GEO database and, more specifically, the compounds that significantly upregulate the expression of HRD1 with a q-value < 0.05 and an absolute log2 fold change > 1 [28].

### Chemical Reagents and Antibodies

We have purchased luteolin and 0.4% trypan blue solution from Wako Pure Chemicals (Osaka, Japan). 6-OHDA hydrobromide was obtained from Sigma-Aldrich (St. Louis, MO, USA), and 3-(4,5-dimethyl-2-thiazolyl)-2,5-diphenyltetrazolium bromide (MTT) was obtained from Dojindo Laboratories (Kumamoto, Japan). All chemicals (Wako Pure Chemicals or Nacalai Tesque Inc., Kyoto, Japan) used in the experiments were either of the highest available grade or of an analytical grade.

Anti-HRD1 (Ab170901) antibody was procured from Abcam (Cambridge, UK). The anti-SEL1L (sc-377,350) and the anti-C/EBP homologous protein (CHOP) (sc-7351) antibodies were obtained from Santa Cruz Biotechnology (Santa Cruz, CA, USA), while the anti-cleaved caspase-3 (Asp175) antibody was purchased from Cell Signaling Technology (Danvers, MA, USA). The anti-β-actin (A2066) antibodies were purchased from Sigma-Aldrich. Finally, the horseradish peroxidase-conjugated goat antirabbit (7074 S) and antimouse (7076 S) secondary antibodies were obtained from Cell Signaling Technology.

### Cell Culture

SH-SY5Y cells, a human neuroblastoma cell line, were cultured in Dulbecco’s modified Eagle’s medium supplemented with 10% fetal bovine serum (Thermo Fisher Scientific, Waltham, USA) in a humidified atmosphere with 5% CO_2_, at 37 °C. Drugs were added to the cells seeded in 24-well plates for the undertaking of the MTT assays or the trypan blue exclusion (TBE) assays, or in 6-well plates for the undertaking of real-time polymerase chain reaction (PCR) or Western blotting.

### RNA Preparation and Reverse Transcription

Total RNA was isolated from the cells by using the QIAshredder kit and the RNeasy Plus Mini Kit (QIAGEN GmbH, Hilden, Germany) according to the manufacturer’s instructions. Moreover, random-primed complementary DNA (cDNA) was prepared from 1 µg of total RNA by using the High-Capacity cDNA Reverse Transcription Kit (Thermo Fisher Scientific) according to the manufacturer’s instructions. cDNA was used as a template for the undertaking of the quantitative real-time PCR.

### Quantitative Real-Time PCR

Quantitative real-time PCR was carried out by using the StepOnePlus Real-Time PCR System (Thermo Fisher Scientific) along with a TaqMan Fast Advanced Master Mix (Thermo Fisher Scientific) for mRNA quantitation. We used probe-primer solutions for mRNA (obtained from Thermo Fisher Scientific) that were specific for *HRD1* (Hs00381211_m1), *SEL1L* (Hs01071406_m1), *CHOP* (Hs00358796_g1), and *18 S rRNA* (Hs99999901_s1). The 18 S rRNA was used as an internal control to normalize the mRNA expression.

### Western Blotting Analysis

We followed the methodology of a previous report [29]. After rinsing with ice-cold phosphate-buffered saline (PBS), the cells were treated with ice-cold lysis buffer (containing 20 mM HEPES, 120 mM NaCl, 5 mM EDTA, 1% Triton X-100, 10% glycerol, 10 mM dithiothreitol, 0.5 mM Phenylmethylsulfonyl fluoride, 1 mM sodium fluoride, and 5 µg/mL leupeptin), and the concentrations of protein were measured by using the Bradford assay. Equal amounts of total protein were separated by employing sodium dodecyl sulfate-polyacrylamide gel electrophoresis, and were then transferred to nitrocellulose blotting membranes. Blocking was conducted at room temperature, for 30 min, in Tris-buffered saline (TBS) with 0.05% Tween 20 containing 5% skim milk (Yukijirushi, Tokyo, Japan), followed by an overnight incubation at 4 °C with primary antibodies against HRD1 (1:1,000), SEL1L (1:500), cleaved caspase-3 (1:1,000), or β-actin (1:5,000) in TBS with 0.05% Tween 20. The appropriate secondary antibodies (antimouse antibodies against SEL1L at a 1:1,000 dilution, and antirabbit antibodies against HRD1, cleaved caspase-3 and β-actin at a 1:5,000 dilution) were used, and the proteins were then visualized by chemiluminescence (ECL Prime or ECL Select; Cytiva, Massachusetts, United States). Blotting images were acquired by ChemiStage CC-16 (KURABO, Osaka, Japan). The protein expression intensity, that was analyzed through the ImageJ software (National Institutes of Health, Bethesda, MD, USA), was corrected by the respective β-actin expression.

### MTT Assay

Cells were incubated with 50 µL of 5 mg/mL MTT solution (final concentration: 0.5 µg/µL) for 20 min at 37 °C. Thereafter, the medium was discarded and the stained cells were lysed in 1 mL of dimethyl sulfoxide. The optical density was measured at 560 nm with a microplate reader (reference wavelength: 630 nm) (infinite M200 PRO; TECAN, Maennedorf, Switzerland).

### TBE Assay

TBE assay was conducted by the methodology of a previous report [22]. Briefly, cells were rinsed with PBS buffer, and trypsinized. The cell suspensions were centrifuged at 200×g for 5 min, and the supernatants were removed. The pellet of the cells was re-suspended in 100 µL PBS buffer, and 70 µL of the cell suspension were dyed with 70 µL of 0.4% trypan blue solution. Cell counts and viability were visually assayed by using a light microscope and hemocytometer.

### RNA Interference

The specific small interfering RNAs (siRNAs) for HRD1 (M-007090–01) and SEL1L (M-004885-01) as well as the corresponding negative scrambled control siRNA (D-001206-14) were procured from Dharmacon (GE Healthcare, Lafayette, CO, USA). The siRNA transfection was performed by using the Lipofectamine RNAiMAX reagent (Invitrogen, MA, USA).

### Statistical Analysis

Quantitative data were presented as mean ± standard error of the mean (SEM). Data were analyzed through Student’s *t*-test or one-way analysis of variance (ANOVA), followed by Dunnett’s two-tailed test or Tukey–Kramer’s two-tailed test. Probability values lower than 0.05 were considered as statistically significant. All statistical analyses were performed by using the IBM SPSS Statistics (version 26; IBM, Armonk, USA) software.

## Results

### Luteolin was Selected as a Compound that Increases Expression of HRD1 by DGB

**Fig. 1:**
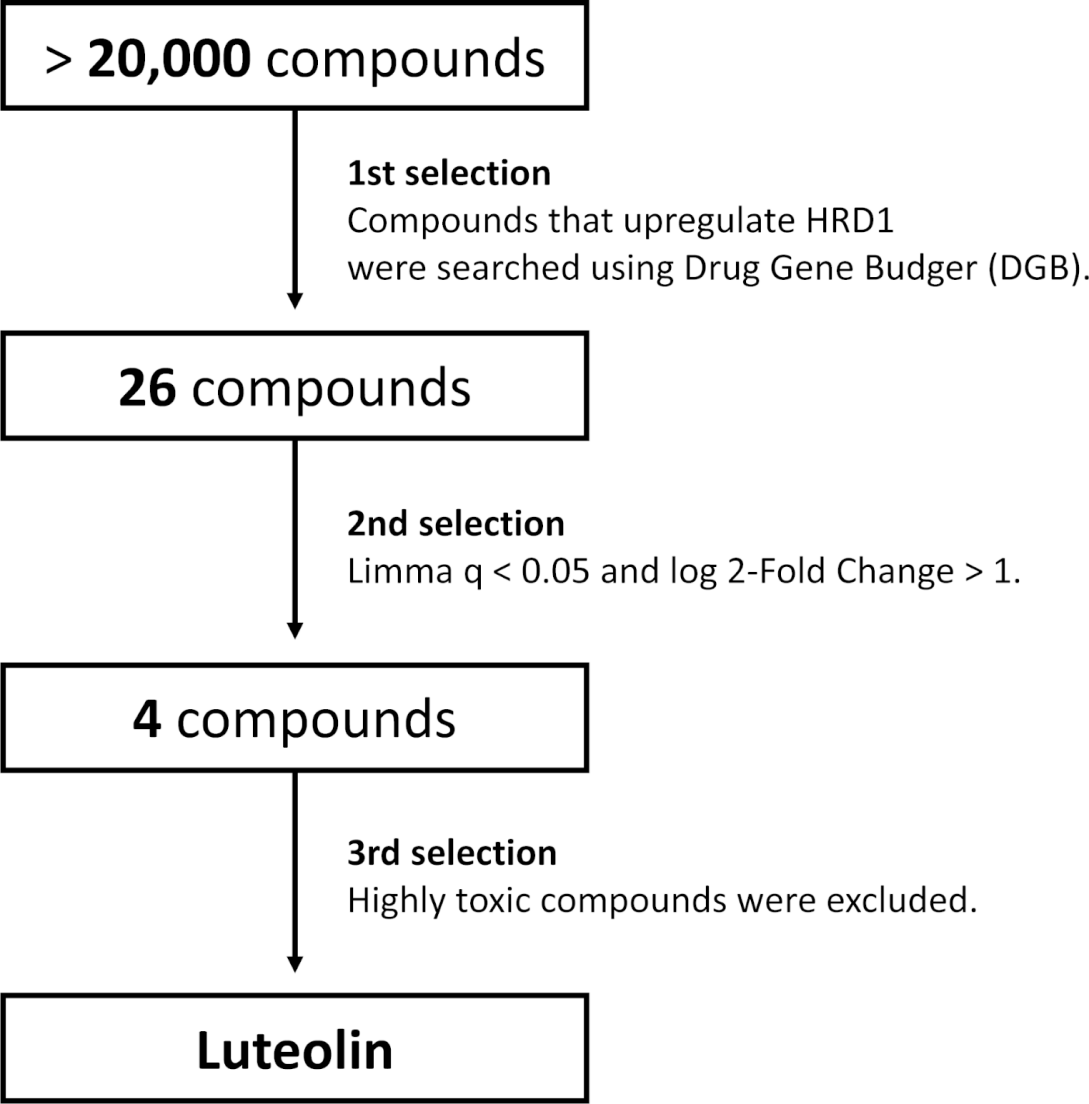
Selection schema for identification of compounds that elevate HRD1 as a therapeutic candidate for PD by DGB. First step: a search was conducted in DGB by using the term “SYVN1”; as a result, 26 compounds were identified as compounds that upregulate the SYVN1/HRD1 pathway. Second step: by using the Limma criteria (q-value < 0.05 and log_2_ fold change > 1), four compounds were selected out of the 26 compounds identified in the first step: doxorubicin, 3,3′,4,4′-tetrachlorobiphenyl, 4-hydroxynonenal, and luteolin. Third step: we excluded the highly toxic compounds; with the exception of luteolin, all other compounds were excluded due to their high toxicity

DGB was utilized to explore the molecules that upregulate the expression of HRD1. Twenty-six compounds were extracted (Supplementary Table 1) as candidates by 1st selection step. And then, the Limma criteria (q-value < 0.05 and log2 fold change > 1) was applied to the 26 candidate compounds [28], and four candidates (namely, doxorubicin, 3,3′,4,4′-tetrachlorobiphenyl, 4-hydroxynonenal, and luteolin) were identified by 2nd selction step. Luteolin was finally selected after excluding the highly toxic compounds among the aforementioned four candidates (Fig. 1).

### A low Concentration of Luteolin Exerted no Cytotoxicity to SH-SY5Y Cells

**Fig. 2:**
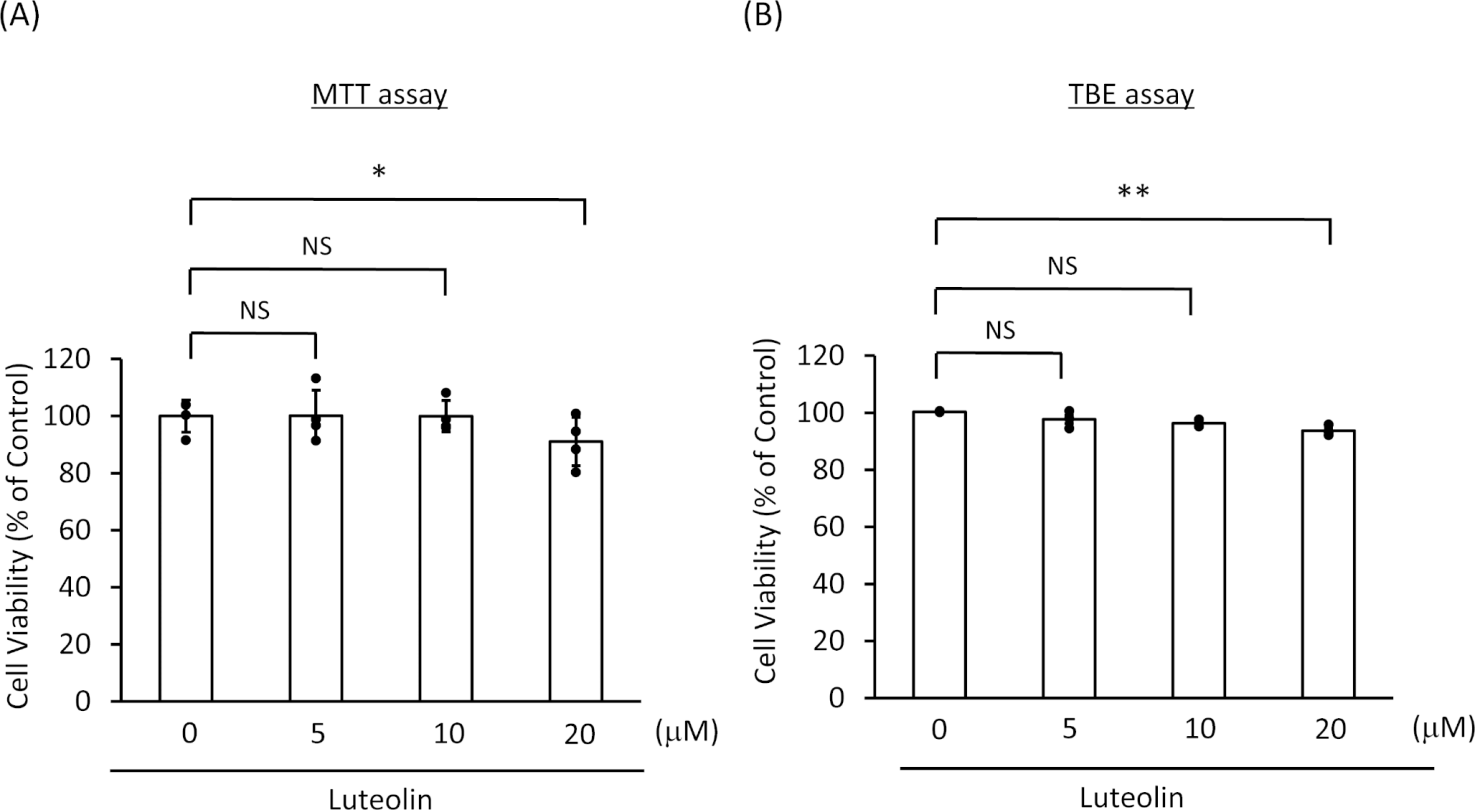
Low concentrations of luteolin were not cytotoxic to SH-SY5Y cells **(A, B)** SH-SY5Y cells were stimulated with 5, 10, or 20 µM luteolin for 24 h. Data on cell viability are expressed as mean ± SEM of four (MTT assay) or three (TBE assay) independent experiments. NS: not significant, *: *p* < 0.05; **: *p* < 0.01; statistical analysis performed *via* one-way ANOVA followed by Dunnett’s *post hoc* test

We examined whether luteolin affects the cell viability of the SH-SY5Y cells by using an MTT and a TBE assay. Figure 2 A and B show that 5 or 10 µM luteolin did not influence the cell viability of the SH-SY5Y cells, while 20 µM luteolin significantly decreased the cell viability (*p* < 0.01 or < 0.05). Based on these findings, the luteolin concentration was fixed at 5 µM in the following experiments.

### Luteolin Increased the HRD1 and SEL1L mRNA and Protein Levels

**Fig. 3:**
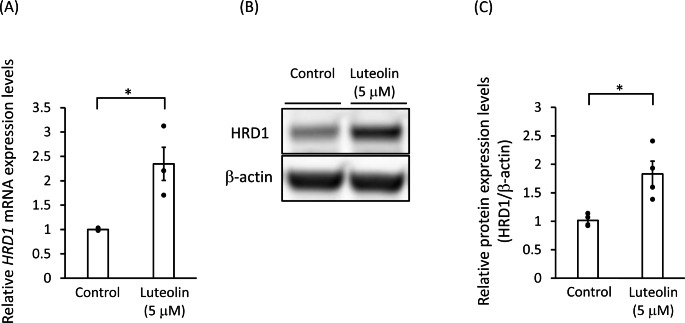
Luteolin increased the HRD1 mRNA and protein levels SH-SY5Y cells were treated with 5 µM luteolin for 24 h. **(A)** The relative expression levels of the *HRD1* mRNA are presented as mean ± SEM of three independent experiments performed in duplicate. *: *p* < 0.05; statistical analysis performed *via* Student’s *t*-test. **(B)** Representative Western blots of HRD1 and β-actin. **(C)** Immunoreactive bands were quantified and expressed as mean ± SEM of four independent experiments. *: *p* < 0.05; statistical analysis performed *via* Student’s *t*-test

**Fig. 4:**
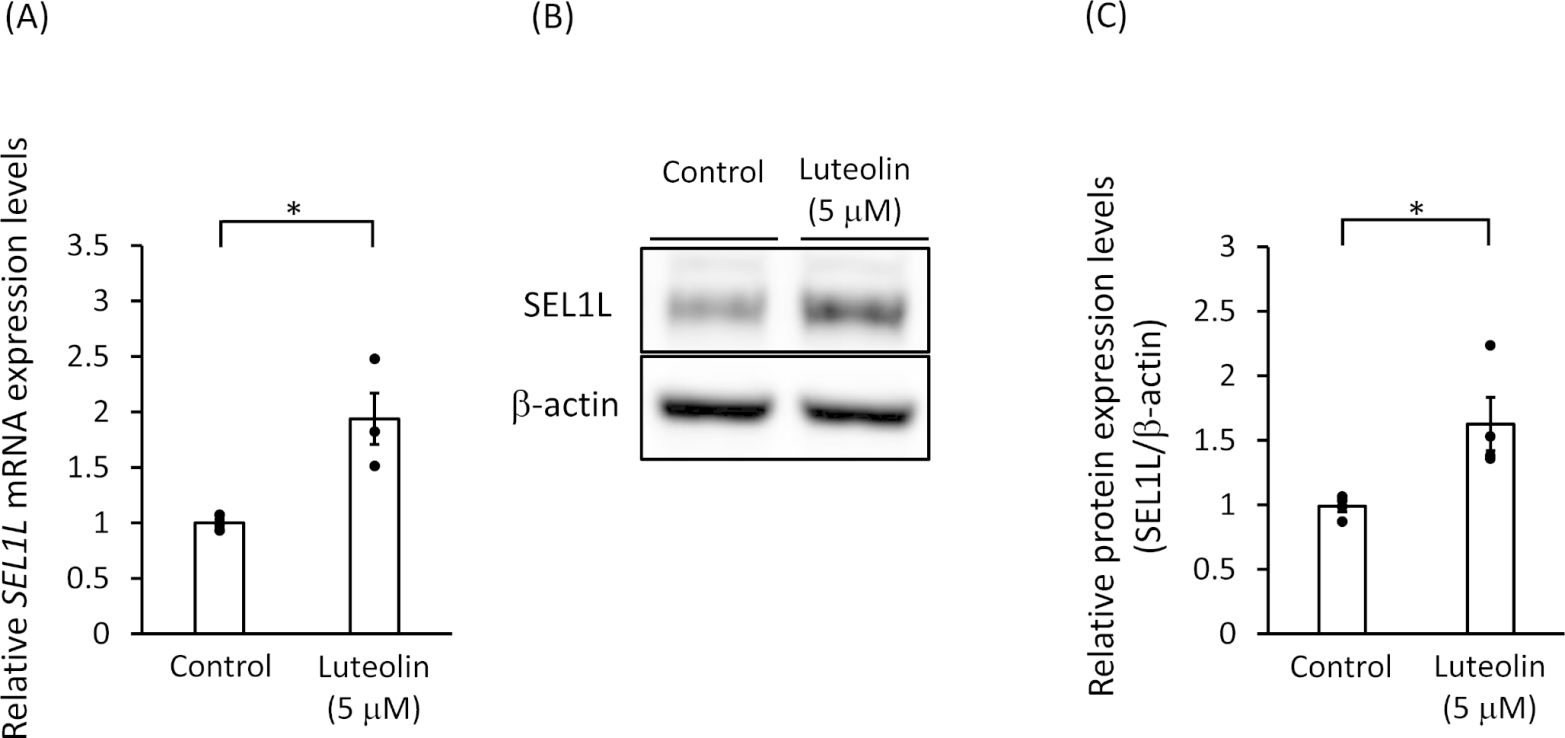
Luteolin increased the SEL1L mRNA and protein levels SH-SY5Y cells were treated with 5 µM luteolin for 24 h. **(A)** The relative expression levels of *SEL1L* mRNA are presented as mean ± SEM of three independent experiments performed in duplicate. *: *p* < 0.05; statistical analysis performed *via* Student’s *t*-test. **(B)** Representative Western blots of SEL1L and β-actin. **(C)** Immunoreactive bands were quantified and expressed as mean ± SEM of four independent experiments. *: *p* < 0.05; statistical analysis performed *via* Student’s *t*-test

We examined whether luteolin can actually upregulate the expression levels of HRD1. A luteolin treatment (5 µM for 24 h) in SH-SY5Y cells significantly increased the levels of the *HRD1* mRNA (Fig. 3A) and the HRD1 protein (Fig. 3B and C). Additionally, the mRNA and the protein levels of SEL1L were also increased by the treatment of luteolin (Fig. 4).

### Luteolin Suppressed the 6-OHDA-Induced Cell Death, Caspase-3 Activation, and ER Stress

**Fig. 5:**
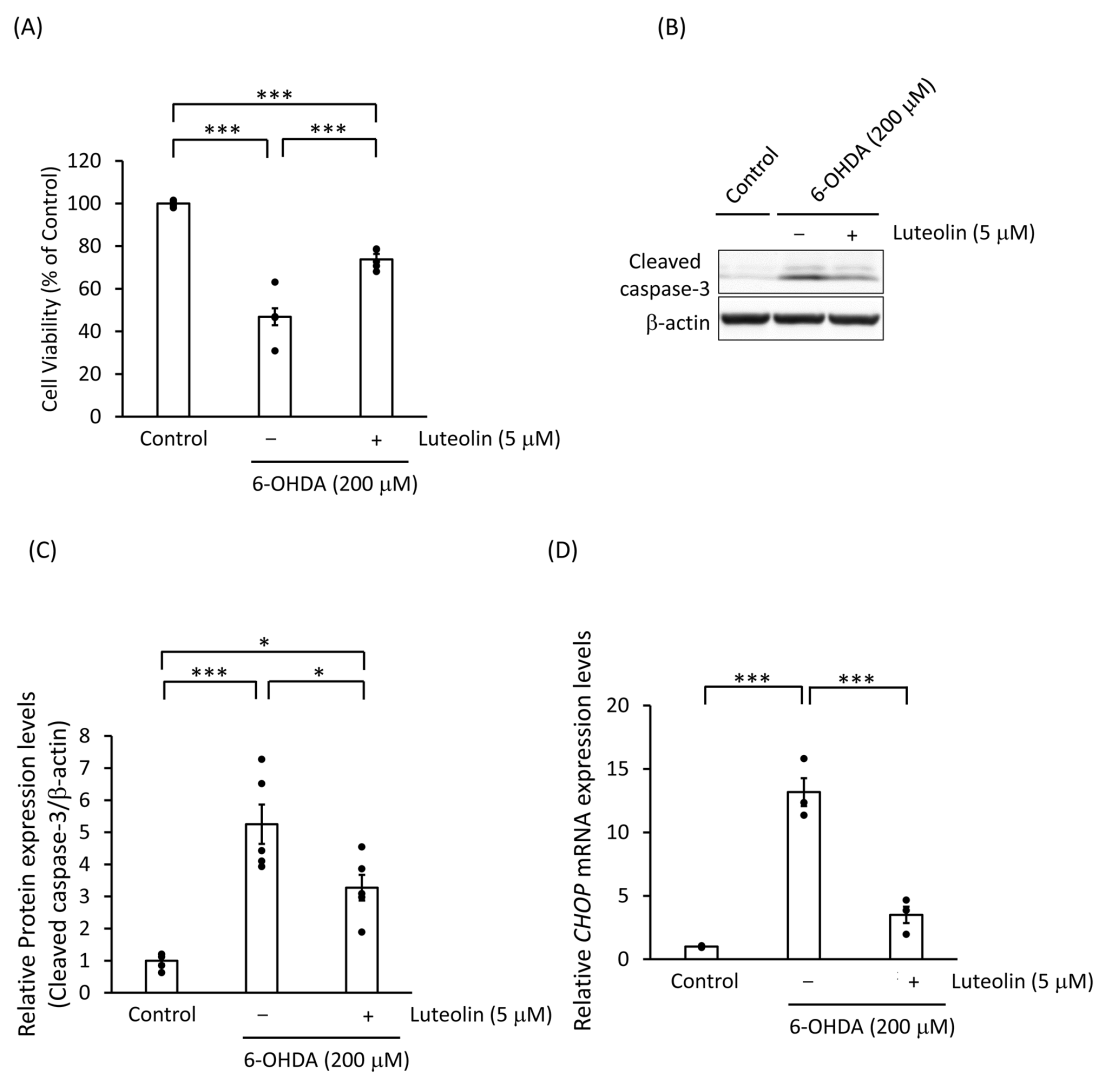
Luteolin suppressed the 6-OHDA-induced cell death, caspase-3 activation, and ER stress response in SH-SY5Y cells SH-SY5Y cells were pretreated with 5 µM luteolin for 2 h prior to a stimulation with 200 µM 6-OHDA for 24 h. **(A)** Data on cell viability are expressed as mean ± SEM of three independent experiments. ***: *p* < 0.001; statistical analysis performed *via* one-way ANOVA followed by Tukey’s *post hoc* test. **(B)** Representative Western blots of cleaved caspase-3 and β-actin. **(C)** Immunoreactive bands were quantified and expressed as mean ± SEM of five independent experiments. *: *p* < 0.05; ***: *p* < 0.001; statistical analysis performed *via* one-way ANOVA followed by Tukey’s *post hoc* test. **(D)** The data of the relative expression levels of *CHOP* mRNA are presented as mean ± SEM of three independent experiments. ***: *p* < 0.001; statistical analysis performed *via* one-way ANOVA followed by Tukey’s *post hoc* test

We subsequently examined whether luteolin can prevent the cell death caused by 6-OHDA in SH-SY5Y cells. As shown in Fig. 5A, luteolin co-treatment with 6-OHDA had significantly lower cytotoxicity compared to 6-OHDA alone. The detection of cleaved caspases is commonly used as a marker of apoptosis [30]; therefore, we detected the levels of cleaved caspase-3, the active form of the proapoptotic protein caspase-3. As shown in Fig. 5B C, luteolin co-treatment with 6-OHDA had significantly lower proten levels of cleaved caspase-3 compared to 6-OHDA alone.

We also analyzed the mRNA levels of *CHOP*; an ER stress marker [31]. 6-OHDA increased the mRNA levels of *CHOP*, while luteolin suppressed these levels (Fig. 5D).

### Luteolin did not Repress the 6-OHDA-Induced Cell Death Under Conditions Involving The Suppression of HRD1 or SEL1L

**Fig. 6:**
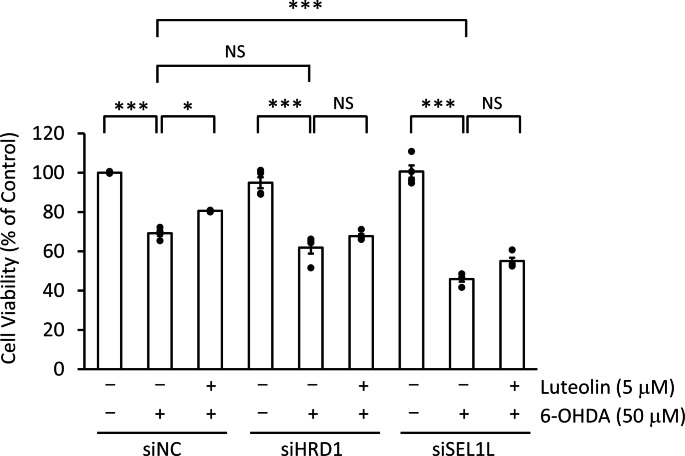
Luteolin did not suppress the 6-OHDA-induced cell death under HRD1- or SEL1L-knockdown conditions SH-SY5Y cells were transfected with the negative control (siNC), the siRNA of HRD1 (siHRD1), or the siRNA of SEL1L (siSEL1L) for 48 h, and were then stimulated with or without 5 µM luteolin and/or 50 µM 6-OHDA for 24 h. Changes in cell viability are expressed as mean ± SEM of four independent experiments. NS: not significant, *: p < 0.05, ***: *p* < 0.001; statistical analysis performed *via* one-way ANOVA followed by Tukey’s *post hoc* test

A possibility mediating HRD1 or SEL1L in cell protective effects of luteolin on 6-OHDA-induced cell death was assessed by using SH-SY5Y cells transiently transfected with HRD1 or SEL1L siRNA. We confirmed that when SH-SY5Y cells were transfected with HRD1 or SEL1L siRNA, expression levels of the respective proteins were suppressed (Supplementary Fig. 1). While luteolin significantly suppressed 6-OHDA-induce cell death when SH-SY5Y cells were transfected with negative control siRNA (siNC), luteolin did not significantly prevent the 6-OHDA-induced death of cells transfected with HRD1 or SEL1L siRNA (Fig. 6).

## Discussion

HRD1 is a ubiquitin ligase involved in ER stress, and SEL1L has been identified as an HRD1 stabilizer [9, 13, 32]. We have previously reported that HRD1 can alleviate the neuronal cell death in familial and nonfamilial PD models [17, 20]. We have further reported that the knockdown of SEL1L by RNA interference also enhances cell death in an in vitro PD model, suggesting an important role for SEL1L in the pathogenesis of PD. [20]. Based on these facts, we hypothesized that the compounds that upregulate the HRD1 expression would prevent the ER stress and suppress the pathogenesis of PD. Icariin, a flavonoid derived from the horny goat weed or *yin yang huo*, is the only known compound that upregulates HRD1 and suppresses the ER stress. However, to date, no other compound (including icariin) has been reported to exert protective effects in PD models [33]. Therefore, we searched for new therapeutic candidates by employing a drug repositioning approach.

Drug repositioning is an approach allowing us to discover new pharmacological effects of already approved drugs. It has recently attracted a lot of attention as the contributions to scientific and technological progress. Advances in microarrays and next-generation sequencers have accelerated the generation of vast amounts of genomic data suitable for drug repositioning studies. Such genomic datasets are easily accessible from public databases such as GEO, CMAP, and LINCS L1000. In this study, we utilized DGB, that integrates these three databases, to search for compounds that upregulate HRD1. Notably, in a previous study that used DGB, prochlorperazine, meclizine, rottlerin, cephaeline, and tretinoin have been identified as candidates for the treatment of papillary thyroid cancer [34].

We searched for compounds that upregulate HRD1 in the DGB. As a result, 26 compounds were extracted only from the GEO database in DGB, and after narrowing them down to those that satisfied the Limma criteria (q-value < 0.05 and log2 fold change > 1), four compounds (namely, doxorubicin, 3,3′,4,4′-tetrachlorobiphenyl, 4-hydroxynonenal, and luteolin) remained. Doxorubicin is a cytotoxic anticancer drug and, therefore, highly toxic, 3,3′,4,4′-tetrachlorobiphenyl is a polychlorinated biphenyl and is known to be highly toxic [35]. Besides, 4-hydroxynonenal is produced by the body and is a causative agent for various diseases [36]. It is known that ER stress occurs when cells are impaired by highly toxic compounds or stress inducers [37, 38]. HRD1 and SEL1L are known as molecules that resist ER stress, and when cells are exposed to ER stress caused by the highly toxic compounds or stress inducers, the expression of HRD1 and SEL1L is elevated to protect them [39]. Thus, it was unsurprising that highly toxic compounds and stress inducers were extracted from the database as molecules that upregulate the expression of HRD1. Therefore, only luteolin was selected as a candidate since we narrowed our focus to compounds with a high safety profile.

Luteolin (3′,4′,5,7-tetrahydroxyflavone) is a natural flavonoid that is present in chrysanthemum flowers, celery, sweet bell peppers, carrots, onion leaves, broccoli, and parsley [40]. Luteolin has been marketed as a dietary supplement in Japan [41]. Luteolin is characterized by multiple bioactivities and neuroprotective effects, exhibits anti-inflammatory activity in microglia [42], and can attenuate the neurotoxicity induced by peroxide [43] or 6-OHDA in cell cultures [44]. Luteolin can cross the blood-brain barrier and has been shown to have anti-amnesic effects against the toxicity of amyloid β protein in mice [45]. There are reports that luteolin suppresses the ER stress [46, 47], while some reports demonstrated that it can trigger ER stress [48, 49]. However, there are no reports of luteolin being involved in the regulation of HRD1 or SEL1L.

Prior studies have reported that luteolin can inhibit cell death and, conversely, that it can enhance cell death [43, 50, 51]. Actually, the reports mentioned above indicate that low concentrations of luteolin can inhibit cell death, while its high concentrations can enhance cell death. Therefore, in order to find a concentration at which luteolin does not affect the cell viability, we added various concentrations of luteolin to SH-SY5Y cells and examined their effects on cell viability. We employed the MTT cell viability test in order to determine cell death; a valid and well-established method. However, there is a drawback in this test: when used with proliferating cells (as in this study), the MTT assay cannot discriminate between cell death and cell growth arrest. In principle, the results generated with this assay could also be attributed to effects on cell growth. Therefore, it is essential to measure the cell viability and to directly determine the number of dead cells (e.g., with the TBE assay or with propidium iodide staining). Accordingly, we have performed the TBE assay under the same experimental conditions, so as to ensure that we detect cell death. The results of the MTT and the TBE assays revealed that high concentrations of luteolin (20 µM or higher) can significantly decrease the cell viability, while low concentrations of luteolin (10 µM or lower) did not affect the SH-SY5Y cell survival. These findings suggest that a treatment concentration of 10 µM or less of luteolin is desirable, which supports previous research [51].

We also confirmed that luteolin alone can increase the HRD1 mRNA and protein levels. Furthermore, since SEL1L is a stabilizing molecule for HRD1, we hypothesized that SEL1L is involved in the luteolin-induced upregulation of HRD1. Subsequently, we confirmed that both the mRNA and the protein levels SEL1L are elevated by luteolin.

Luteolin inhibited the 6-OHDA-induced cell death. Furthermore, present study herein demonstrated that luteolin suppresses the 6-OHDA-induced apoptosis (from a biochemical perspective) by verifying the increase in cleaved caspase-3. In addition, luteolin was shown to suppress a 6-OHDA-induced increase of *CHOP* mRNA, thereby indicating that luteolin inhibits ER stress. However, luteolin does not entirely prevent a 6-OHDA-induced cell death, and this finding correlates with data obtained from the MTT assay and the study of cleaved caspase-3. We hypothesized that this was because 6-OHDA is known to trigger various toxicity mechanisms (oxidative stress, mitochondrial insults, etc.) apart from ER stress, and although the luteolin-induced upregulation of HRD1 can suppress the 6-OHDA-induced ER stress, luteolin might not be able to suppress the other mechanisms of toxicity [52, 53].

In Fig. 5, the cells were treated with 200 µM of 6-OHDA, whereas in the experiment in Fig. 6, the cells were treated with only 50 µM, thus resulting in different cell survival rates. When SH-SY5Y cells are transfected using Lipofectamine RNAiMAX, they are more susceptible to 6-OHDA stimulation than the non-transfected cells. Therefore, we performed the experiments shown in Fig. 6 with lower concentrations of 6-OHDA. Furthermore, as shown in Fig. 6, although there was no significant difference in cell viability after exposure to 6-OHDA in cells transfected with siNC and cells transfected with siHRD1, cell viability was significantly lower in cells transfected with siSEL1L compared to cells transfected with siNC after 6-OHDA exposure. We have previously reported that when SEL1L was suppressed by RNA interference, 6-OHDA-induced cell death was enhanced [20]. The current results were consistent with previous reports. In Fig. 6, we also demonstrated that luteolin significantly suppressed 6-OHDA-induced cell death in cells transfected with siNC. However, luteolin did not suppress 6-OHDA-induced cell death when HRD1 or SEL1L expression was suppressed by RNA interference in SH-SY5Y cells. This result confirmed that luteolin suppresses 6-OHDA-induced cell death via the activation of HRD1 or SEL1L.

It has been known that HRD1 predominantly depends on the inositol requiring 1 alpha (IRE1α)-X-box binding protein 1 (XBP1) pathway, and that SEL1L is dependent on the activating transcription factor 6 alpha (ATF6α) pathway [39]. The *XBP1* mRNA is spliced by IRE1α and acts as a transcription factor [54]. ATF6α is cleaved by site 1 protease and site 2 protease, while the ATF6α N-terminus [ATF6α (N)] acts as a transcription factor [55]. The ATF6α pathway is associated with both the HRD1 and the SEL1L because the ATF6α (N) increases the production of the *XBP1* mRNA [54]. Preliminarily data show that luteolin can reduce the full length of the ATF6α without increasing the ER stress marker CHOP expression, and can also cause a splicing of the *XBP1* mRNA (Supplementary Fig. 2). Through these findings, we propose that luteolin activates the IRE1-XBP1 pathway and ATF6α without inducing ER stress, which in turn increases the expression levels of downstream targets HRD1 and SEL1L. Subsequently, activated HRD1 and SEL1L suppress ER stress caused by 6-OHDA, and may ultimately protect against neuronal cell death, however, further experiments are needed to investigate this hypothesis.

In conclusion, this study has revealed that luteolin can suppress the 6-OHDA-induced neuronal cell death *via* an upregulation of the expression of HRD1 and SEL1L. As HRD1 and SEL1L are critical molecules for neurodegenerative disorders such as PD, luteolin might be a therapeutic drug candidate for the treatment of PD and other neurodegenerative disorders whose neuropathology implicates the ER stress.

### Electronic Supplementary Material

Below is the link to the electronic supplementary material.

Supplementary Material 1

## Data Availability

Publicly available datasets were analyzed in this study. This data can be found here: https://maayanlab.cloud/DGB/.

## Abbreviations

6-OHDA: 6-hydroxydopamine
ANOVA: Analysis of variance
ATF6α: Activating transcription factor 6 alpha
ATF6α (N): ATF6α N-terminus
cDNA: Complementary DNA
CHOP: C/EBP homologous protein
Cmap: Connectivity Map
DGB: Drug Gene Budger
ER: Endoplasmic reticulum
ERAD: ER-associated degradation
GEO: Gene Expression Omnibus
HMG-CoA: 3-hydroxy-3-methylglutaryl-coenzyme A
HRD1: HMG-CoA reductase degradation 1
IRE1α: Inositol requiring one alpha
LINCS: Library of Integrated Network-Based Cellular Signatures
MTT: 3-(4,5-dimethyl-2-thiazolyl)-2,5-diphenyltetrazolium bromide
Pael-R: Parkin-associated endothelin receptor-like receptor
PBS: Phosphate-buffered saline
PCR: Polymerase chain reaction
PD: Parkinson’s disease
SEL1L: Suppressor/enhancer Lin12 1-like
SEM: Standard error of the mean
siRNA: Small interfering RNA
TBE: Trypan blue exclusion
UPR: Unfolded protein response
XBP1: X-box binding protein 1
