# Supplementary material for: Luteolin Protects Against 6-Hydoroxydopamine-Induced Cell Death via an Upregulation of HRD1 and SEL1L
Source: Neurochem Res. 2023 Aug 26;49(1):117–28. doi: 10.1007/s11064-023-04019-2 (PMC10776467; doi:10.1007/s11064-023-04019-2)
Supplement: Supplementary file 1 — Supplementary Material 1 [file 11064_2023_4019_MOESM1_ESM.docx]

**Luteolin protects against 6-hydoroxydopamine-induced cell death *via* an upregulation of HRD1 and SEL1L**

Hiroki Nishiguchi^1^, Tomohiro Omura^1, *^, Ayaka Sato^2^, Yumi Kitahiro^1^, Kazuhiro Yamamoto^1^, Junichi Kunimasa^2^, Ikuko Yano^1^

^1^Department of Pharmacy, Kobe University Hospital, 7-5-2 Kusunoki-cho, Chuo-ku, Kobe, 650-0017, Japan

^2^Education and Research Center for Clinical Pharmacy, Kobe Pharmaceutical University, 4-19-1, Motoyama Kitamachi, Higashinada-ku, Kobe, 658-8558, Japan

**Support or grant information:**

This work was supported by JSPS KAKENHI (grant numbers JP 17K08444 and 20K07154) and JST SPRING (grant number JPMJFS2126).

*Corresponding author: Tomohiro Omura, PhD

Department of Pharmacy, Kobe University Hospital, 7-5-2 Kusunoki-cho, Chuo-ku, Kobe 650-0017, Japan

Phone: +81-78-382-6641

E-mail: [omurat@med.kobe-u.ac.jp](mailto:omurat@med.kobe-u.ac.jp)

**

Supplementary Figure 1.** HRD1 or SEL1L protein expression levels were suppressed through the transfection of each siRNA in SH-SY5Y cells.

SH-SY5Y cells were transfected with negative control siRNA (siNC), siRNA against HRD1 (siHRD1), or siRNA against SEL1L (siSEL1L) for 48 h. Representative Western blots of HRD1, SEL1L and β-actin.

**

Supplementary Figure 2.** Luteolin activated the *XBP1* mRNA and the ATF6α protein.

SH-SY5Y cells were stimulated with 5 µM luteolin for 24 h. ER stress inducers thapsigargin (5 μM) and tunicamycin (5 μg/mL) were used as positive controls for the activation of the ATF6α protein and the *XBP1* mRNA. **(A)** Representative Western blots of ATF6α-full length (sc-166659), CHOP, and β-actin. **(B)** Immunoreactive bands were quantified and expressed as mean ± SEM of three independent experiments. ***: *p* < 0.001; statistical analysis performed *via* Student’s *t*-test. **(C)** After the reverse transcription of the total cellular mRNA, a PCR was performed by using a MiniAmp Plus Thermal Cycler (Thermo Fisher Scientific) for 35 cycles in order to amplify the cDNA for *XBP1* and *β-actin*. Primers were purchased from Eurofins Genomics (Tokyo, Japan), with TTGCTGAAGAGGAGGCGGAAG as the forward primer and GGTCCAAGTTGTCCAGAATGC as the reverse primer for *XBP1*, and with CTGTCTGGCGCACCACCAT as the forward primer and GCAACTAAGTCATAGTCCGC as the reverse primer for *β-actin*. The amplification was carried out by an initial denaturing step at 94°C for 3 min, followed by 35 cycles at 94°C for 30 sec, 60°C for 30 sec, and 72°C for 30 sec, and a final extension step at 72°C for 10 min. The amplified DNA was separated by electrophoresis in a 7.5% acrylamide gel and Tris-borate-EDTA buffer. Fragments of unspliced (*XBP1-u*) and spliced (*XBP1-s*) XBP1 were detected as 210-bp and 184-bp nucleotides, respectively, and *β-actin* was detected as 254-bp. *β-Actin* was used as a loading control. Representative images are provided from at least three independent experiments.

**Supplementary Table 1.** The list of compounds which upregulate HRD1 extracted from the Drug Gene Budger.

|  | **Drug Name** | **CREEDS**  **ID** | **GEO**  **ID** | **q-value** | **Log_2_ (Fold Change)** |
| --- | --- | --- | --- | --- | --- |
| **1** | R1881 | drug:3048 | GSE22606 | 1.32344E-05 | 0.734957 |
| **2** | Doxorubicin^*^ | drug:3179 | GSE58074 | 2.20359E-05 | 1.09024 |
| **2** | Doxorubicin^*^ | drug:3180 | GSE58074 | 9.19692E-05 | 1.35479 |
| **3** | Vx^*^ | drug:3376 | GSE33606 | 0.000235748 | 0.504659 |
| **3** | Vx^*^ | drug:3345 | GSE33606 | 0.00367167 | 0.289713 |
| **4** | Sulforaphane | drug:3445 | GSE28813 | 0.000481915 | 0.959335 |
| **5** | Cadmium | drug:3402 | GSE9951 | 0.000159046 | 0.206697 |
| **6** | Celecoxib | drug:2501 | GSE54657 | 0.00202067 | 0.0808803 |
| **7** | Dasatinib | drug:3306 | GSE59357 | 0.000369876 | 0.252031 |
| **8** | R1881 | drug:3049 | GSE22606 | 0.00194924 | 0.278615 |
| **9** | Diclofenac | drug:3053 | GSE54255 | 0.000519524 | 0.36993 |
| **10** | Phorbol 12-myristate 13-acetate  (pma) | drug:3246 | GSE45417 | 0.00137649 | 0.644333 |
| **11** | Cisplatin^*^ | drug:3152 | GSE47856 | 0.00109946 | 0.45049 |
| **11** | Cisplatin^*^ | drug:3143 | GSE47856 | 0.0472529 | 0.427889 |
| **12** | 3,3',4,4'-tetrachlorobiphenyl | drug:3273 | GSE6878 | 0.00823687 | 1.09382 |
| **13** | Imatinib | drug:2526 | GSE22433 | 0.00245209 | 0.377689 |
| **14** | Anastrozole | drug:3087 | GSE33658 | 0.0474844 | 0.220547 |
| **15** | Nitric oxide | drug:3181 | GSE13887 | 0.00389718 | 0.27213 |
| **16** | 4-hydroxynonenal | drug:3082 | GSE2397 | 0.00765351 | 1.09032 |
| **17** | Thapsigargin | drug:3236 | GSE19519 | 0.0192114 | 0.709334 |
| **18** | Resveratrol | drug:3501 | GSE25412 | 0.0172303 | 0.415437 |
| **19** | Luteolin | drug:3473 | GSE53180 | 0.0425411 | 1.07593 |
| **20** | Estradiol | drug:3203 | GSE12446 | 0.0261111 | 0.242846 |
| **21** | Alfacalcidol | drug:3362 | GSE15947 | 0.0487523 | 0.116629 |
| **22** | Sapphyrin pci-2050  (1.25 &icirc;&frac14;m) | drug:3101 | GSE6400 | 0.0164823 | 0.248073 |
| **23** | Adenosine triphosphate | drug:3220 | GSE30903 | 0.0330875 | 0.218993 |
| **24** | Bisphenol a | drug:2663 | GSE17624 | 0.0268643 | 0.426899 |
| **25** | Bpde | drug:3379 | GSE19510 | 0.0267315 | 0.0822352 |
| **26** | Vemurafenib | drug:2495 | GSE42872 | 0.0441972 | 0.123833 |
| ^*^As two types of data existed when accommodated in CREEDS,  there are compounds with the same GEO ID but different CREEDS IDs. | | | | | |
